# A Rare Presentation of Subacute Constrictive Pericarditis Secondary to Rhinovirus Infection: A Case Report

**DOI:** 10.7759/cureus.104481

**Published:** 2026-03-01

**Authors:** Teddy A Teddy, Edidiong Okon-Ben, America Silva, Angelo MessinaAlvarez, Luis Afonso

**Affiliations:** 1 Internal Medicine, Wayne State University Detroit Medical Center, Detroit, USA; 2 Cardiology, Wayne State University Detroit Medical Center, Detroit, USA

**Keywords:** annulus reversus, cardiac magnetic resonance imaging, constrictive pericarditis, echocardiography, pericardial effusion, rhinovirus, subacute pericarditis, ventricular interdependence, viral pericarditis

## Abstract

Constrictive pericarditis is an uncommon but important complication of pericardial inflammation in which a stiff, scarred pericardium restricts normal ventricular filling. It is most often related to prior surgery, radiation, tuberculosis, or idiopathic causes, while viral causes rarely progress to constrictive physiology. Patients typically present with nonspecific symptoms such as shortness of breath and signs of right-sided heart failure, which can make diagnosis challenging. We describe a case of subacute constrictive pericarditis associated with rhinovirus infection after other common causes were excluded. This case highlights the need to consider uncommon viral etiologies and perform a thorough evaluation when assessing patients with suspected constrictive pericarditis.

The patient was a 49-year-old woman with no significant past medical history who presented with one month of pleuritic chest pain, progressive dyspnea, orthopnea, palpitations, and subjective fevers. Physical examination revealed tachycardia, muffled heart sounds, and a pericardial friction rub. Laboratory evaluation demonstrated markedly elevated inflammatory markers and an increased serum cardiac troponin level. CT of the chest showed a moderate pericardial effusion. Transthoracic echocardiography (TTE) revealed a circumferential organized pericardial effusion, exaggerated respiratory variation in mitral and tricuspid inflow velocities, annulus reversus, and septal bounce, findings compatible with early constrictive physiology. An extensive infectious and rheumatologic workup was unrevealing, with the exception of a positive rhinovirus polymerase chain reaction result. Treatment with nonsteroidal anti-inflammatory therapy, colchicine, and corticosteroids led to complete clinical and echocardiographic resolution.

Rhinovirus infection may, in rare instances, lead to subacute constrictive pericarditis. Early recognition through echocardiography and timely initiation of anti-inflammatory therapy may result in complete reversibility and reduce the risk of progression to chronic constrictive pericarditis that necessitates surgical intervention.

## Introduction

Constrictive pericarditis is a rare condition in the general population, with an estimated incidence of approximately 0.2 to 0.4 per 100,000 persons per year. It develops in about 1 to 2% of acute pericarditis cases and is characterized by impaired ventricular filling due to a rigid, noncompliant pericardium, resulting in reduced cardiac output and clinical signs of diastolic heart failure [1]. Although historically considered a chronic and irreversible disease, constrictive pericarditis is now recognized as a spectrum disorder that includes an early inflammatory and potentially reversible phase. Identification of this subacute form is essential, as timely medical therapy can prevent progression to permanent fibrotic constriction requiring surgical pericardiectomy.

The etiologic profile of constrictive pericarditis has changed over time. In developed countries, idiopathic pericarditis, prior cardiothoracic surgery, and mediastinal radiation therapy account for the majority of cases, whereas tuberculosis remains the predominant cause in endemic regions [2]. Viral infections are a leading cause of acute pericarditis, yet progression from viral pericarditis to constrictive physiology is exceptionally rare [3]. When viral etiologies are identified, enteroviruses, adenoviruses, and influenza viruses are most commonly implicated [4,5].

Rhinovirus is a picornavirus responsible for the majority of upper respiratory tract infections worldwide. Despite its widespread prevalence, rhinovirus is rarely linked to clinically significant cardiac involvement. Reports of rhinovirus-associated myocarditis or pericarditis are limited, and progression to constrictive pericarditis is extremely uncommon, with only isolated cases documented [4,5]. This rarity can contribute to diagnostic delays, as clinicians may not readily associate a common respiratory virus with severe pericardial disease. Subacute constrictive pericarditis presents a diagnostic challenge due to its insidious onset and clinical overlap with other causes of diastolic heart failure, particularly restrictive cardiomyopathy [6,7]. Clinical manifestations often include progressive dyspnea, orthopnea, chest discomfort, and fatigue, while classic signs of advanced constriction, such as marked jugular venous distention or peripheral edema, may be absent during the early stages. As a result, imaging plays a central role in establishing the diagnosis.

Transthoracic echocardiography (TTE) remains the cornerstone of evaluation, enabling the assessment of pericardial morphology and the hemodynamic consequences of pericardial constraint. Key echocardiographic features include ventricular interdependence with exaggerated respiratory variation in atrioventricular inflow velocities, paradoxical interventricular septal motion referred to as septal bounce, and preserved longitudinal myocardial relaxation evaluated by tissue Doppler imaging (TDI) [8]. The finding of annulus reversus, defined as higher septal than lateral mitral annular velocities on TDI, is particularly useful for distinguishing constrictive pericarditis from restrictive cardiomyopathy and has high diagnostic specificity [9].

We report a rare case of subacute constrictive pericarditis in a previously healthy woman, attributed to a rhinovirus infection, emphasizing the diagnostic role of echocardiography and the favorable outcomes possible with early anti-inflammatory therapy.

## Case presentation

The patient was a 49-year-old woman with no significant past medical history who presented with one month of sharp chest pain, worsening shortness of breath, difficulty breathing while lying flat, palpitations, and reported fevers. Laboratory evaluation demonstrated leukocytosis with a white blood cell count of 16.8 x 10³/μL and mild hypokalemia (Table 1). Inflammatory markers were markedly elevated, including a C-reactive protein level of 203.4 mg/L and an erythrocyte sedimentation rate of 58 mm/hr, indicating an increased inflammatory burden and complicated pericarditis (Table 2). Cardiac troponin I was elevated to 40 ng/mL, consistent with myocardial involvement and supporting a diagnosis of myopericarditis. Electrocardiography demonstrated sinus tachycardia without ST-segment elevation, PR segment depression, or evidence of ischemic changes.

**Table 1:** Initial hematologic and metabolic evaluation BUN: blood urea nitrogen; GFR: glomerular filtration rate

Investigations	Test	Value	Reference range
Basic metabolic panel	Sodium (mmol/L)	139	135–145
	Potassium (mmol/L)	3.8	3.5–5.0
	Chloride (mmol/L)	103	98–107
	CO₂/bicarbonate (mmol/L)	24	22–29
	Anion gap	12	8–16
	Glucose (mg/dL)	90	70–99
	Calcium (mg/dL)	9.7	8.6–10.2
	Magnesium (mg/dL)	2.0	1.7–2.2
	Phosphorus (mg/dL)	2.9	2.5–4.5
Renal function	BUN (mg/dL)	10	7–20
	Creatinine (mg/dL)	0.67	0.6–1.3
	Estimated GFR (mL/min/1.73m²)	>60	>60
Full blood count	White blood cells (×10⁹/L)	16.3	4.0–11.0
	Hemoglobin (g/dL)	13.3	12–16 (female)/13.5–17.5 (male)
	Hematocrit (%)	40.2	36–46 (female)/41–53 (male)
	Red blood cells (×10⁶/µL)	4.04	4.2–5.4
	Platelets (×10⁹/L)	412	150–400

**Table 2:** Laboratory evaluation excluding other infectious and rheumatologic causes of constrictive pericarditis BNP: B-type natriuretic peptide; ESR: erythrocyte sedimentation rate; TSH: thyroid-stimulating hormone; ANCA-IFA: antineutrophil cytoplasmic antibodies-indirect immunofluorescence assay; PCR: polymerase chain reaction; COVID-19: coronavirus disease 2019; RSV: respiratory syncytial virus; HIV: human immunodeficiency virus

Investigations	Test	Value	Reference range
Cardiac markers	High-sensitivity troponin (ng/L)	40	<14
	BNP (pg/mL)	36	<100
	D-dimer (µg/mL FEU)	0.4	<0.5
Inflammatory markers	C-reactive protein (mg/L)	203.4	<10
	ESR (mm/hr)	58	<20
Thyroid function	TSH (µIU/mL)	2.1	0.4–4.5
Rheumatologic workup	ANCA-IFA	None detected	Negative
	Anti-dsDNA	None detected	Negative
	Complement C3 (mg/dL)	170	90–180
	Complement C4 (mg/dL)	30	10–40
	Rheumatoid factor (IU/mL)	7	<14
	Myeloperoxidase Ab	2.05	<3.5
	Serine protease 3 Ab	2	<20
Infectious workup	Rhinovirus PCR	Detected	Not detected
	COVID-19 PCR	Not detected	Not detected
	Influenza A/B	Not detected	Not detected
	RSV	Not detected	Not detected
	HIV Ag/Ab	Nonreactive	Nonreactive
	QuantiFERON-TB Gold	Negative	Negative

**Figure 1:**
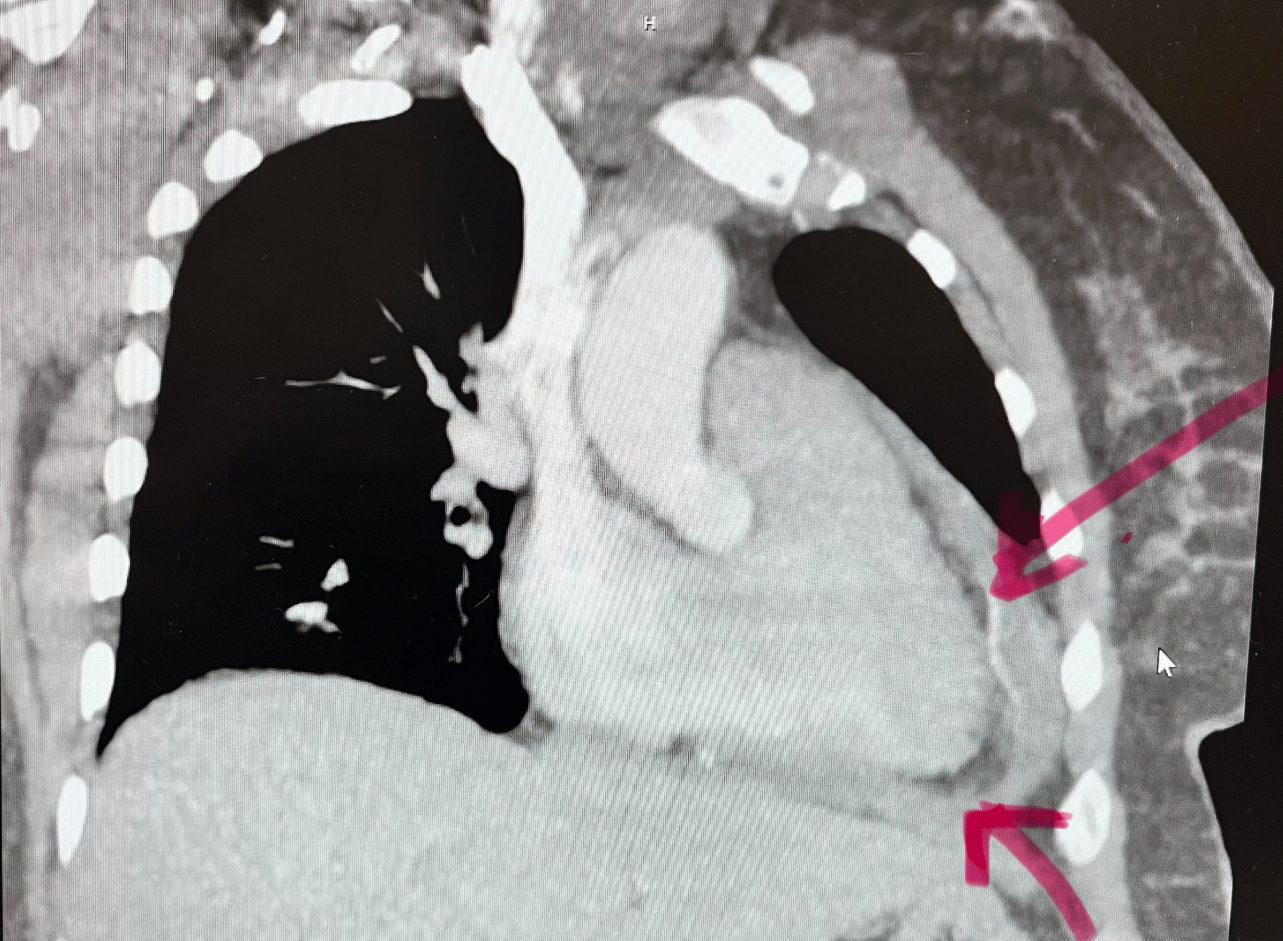
CT thorax showing pleural effusions with associated pericardial thickening CT: computed tomography

CT of the chest was performed to further evaluate the cardiac findings and to exclude pulmonary pathology. The study demonstrated a moderate circumferential pericardial effusion with associated pericardial thickening, as well as a small left-sided pleural effusion. There was no evidence of pulmonary embolism (Figure 1).

**Figure 2:**
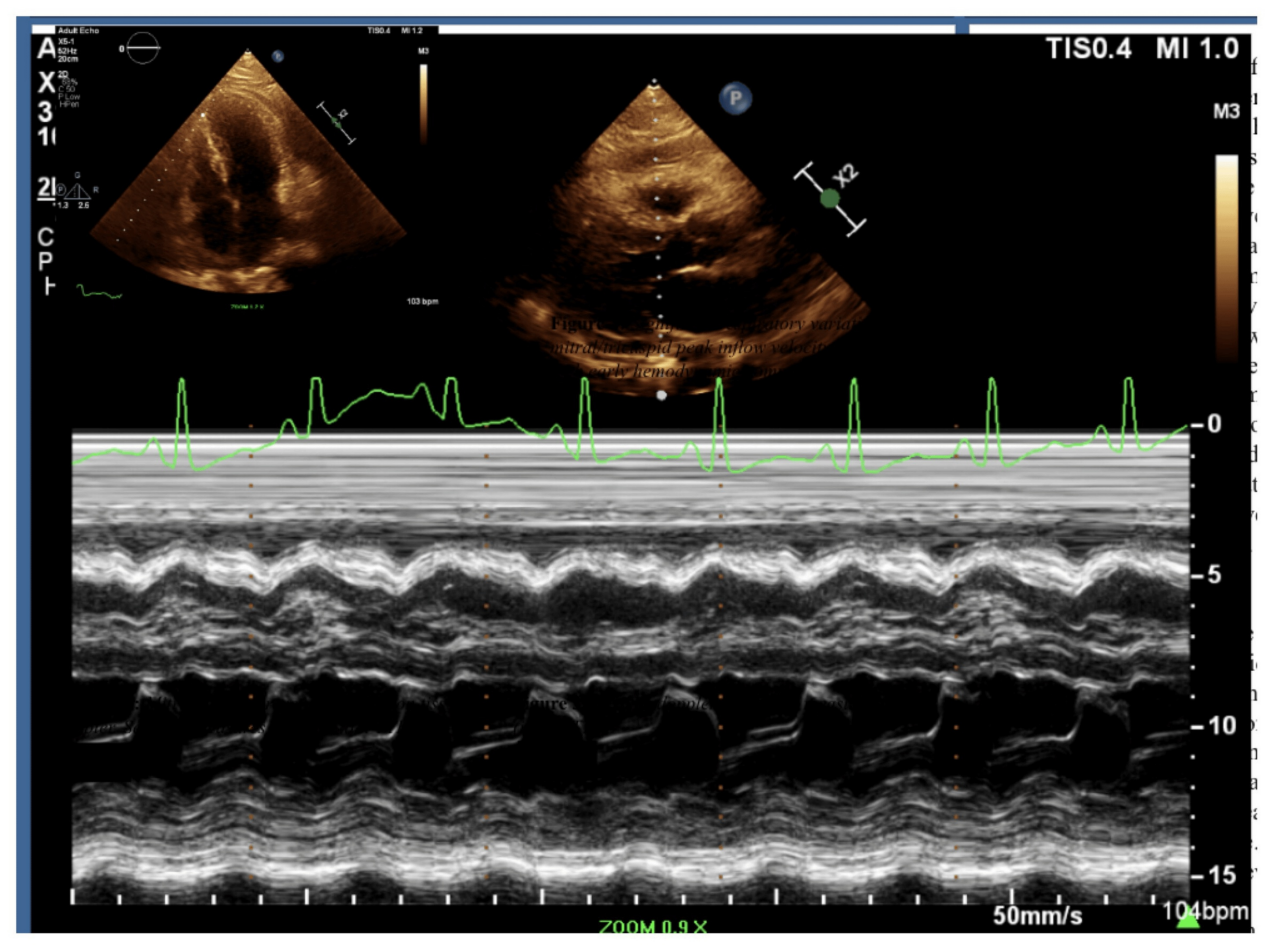
Pulsed wave Doppler assessment of mitral inflow velocities demonstrating marked respiratory variation (>25%) The finding is consistent with ventricular interdependence and early constrictive physiology on initial echocardiography

Subsequent transthoracic echocardiography revealed a moderate circumferential pericardial effusion with an organized appearance and associated pericardial thickening, findings consistent with active pericardial inflammation. Left ventricular systolic function was preserved, and there was no evidence of overt tamponade physiology. Doppler echocardiographic assessment demonstrated exaggerated respiratory variation in transmitral and transtricuspid inflow velocities, reflecting marked ventricular interdependence and early hemodynamic compromise consistent with constrictive physiology (Figure 2).

**Figure 3:**
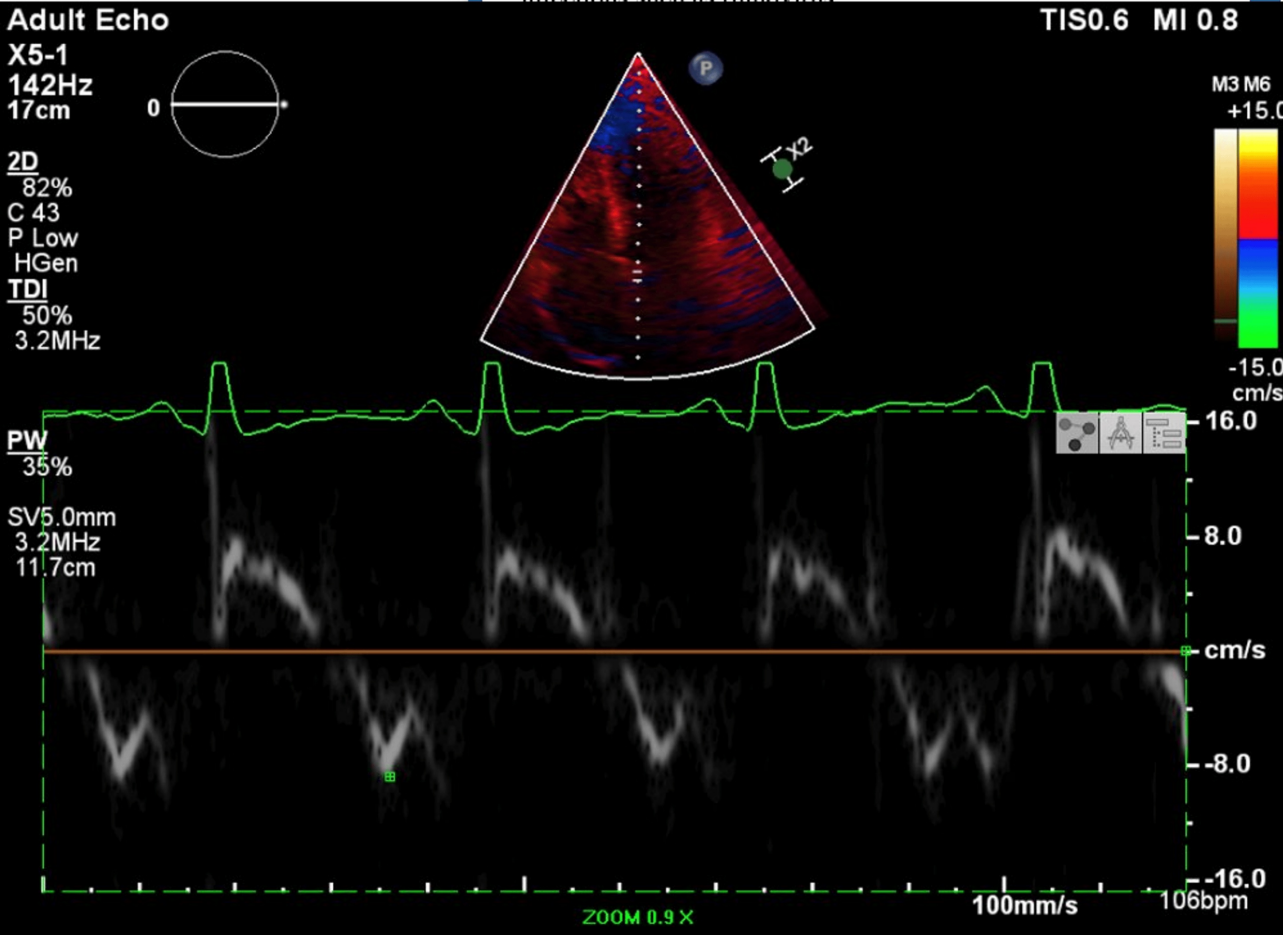
Tissue Doppler imaging from the apical four-chamber view at the septal mitral annulus The early diastolic velocity (e') measures approximately 10 cm/s

**Figure 4:**
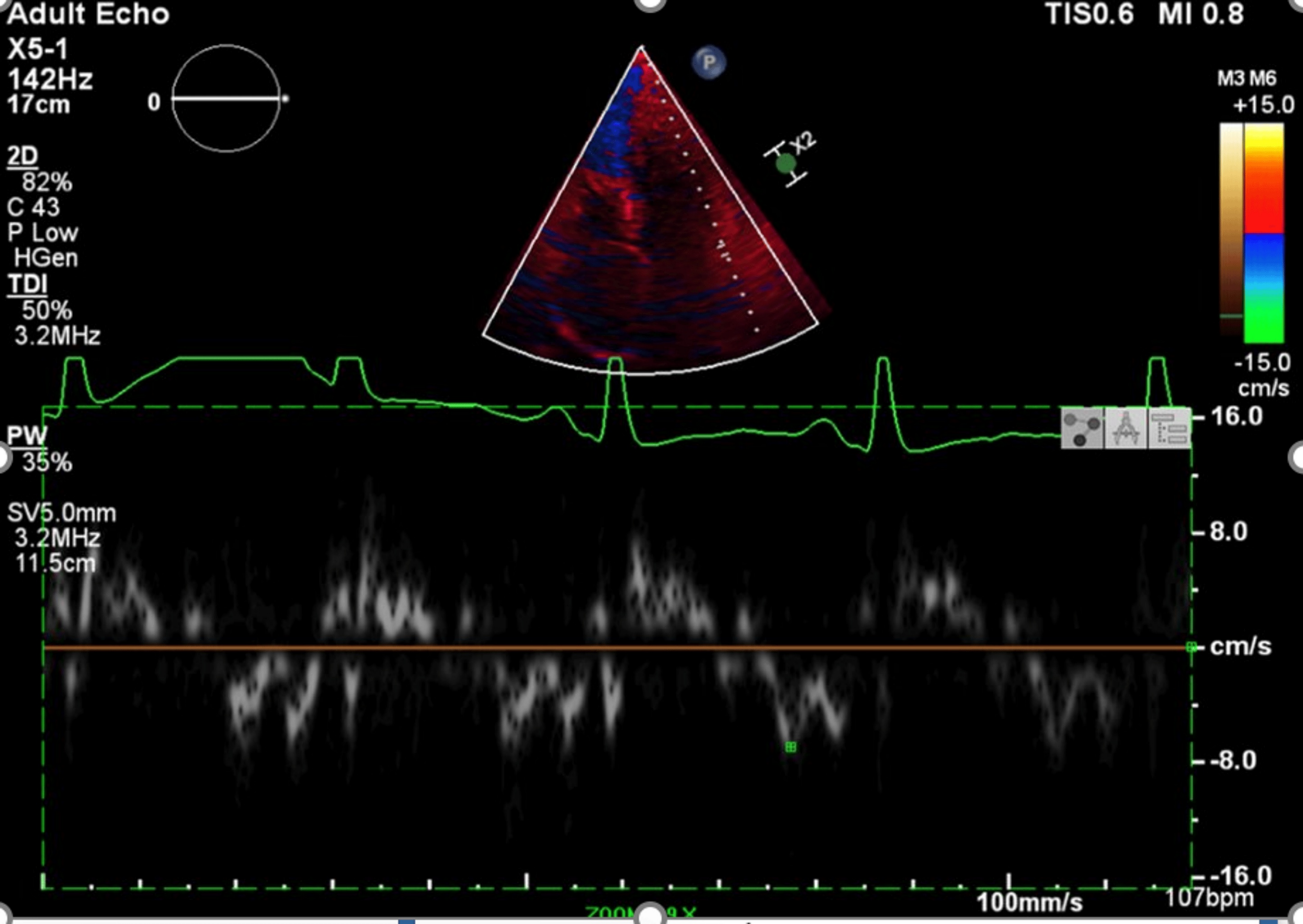
Tissue Doppler imaging from the apical four-chamber view at the lateral mitral annulus The early diastolic velocity (e') measures approximately 8 cm/s. The septal e' velocity (10 cm/s) exceeding the lateral e' velocity (8 cm/s) demonstrates annulus reversus, consistent with constrictive pericarditis

Tissue Doppler imaging revealed preserved longitudinal myocardial relaxation. Notably, the septal mitral annular e' velocity exceeded the lateral e' velocity, a finding known as annulus reversus. This pattern is highly suggestive of constrictive pericarditis and helps distinguish it from restrictive cardiomyopathy (Figures 3, 4).

Two-dimensional imaging demonstrated paradoxical interventricular septal motion during early diastole, commonly referred to as septal bounce, providing further evidence of pericardial constraint. Inferior vena cava size and collapsibility remained within normal limits, consistent with early or subacute constriction rather than advanced disease. The patient was admitted for further evaluation and management. High-dose ibuprofen and colchicine were initiated in accordance with guideline-recommended therapy for acute pericarditis. Given the severity of symptoms, markedly elevated inflammatory markers, a positive rhinovirus test, and echocardiographic evidence of constrictive physiology, intravenous methylprednisolone was started to target the inflammatory component of the constriction and was later transitioned to oral prednisone.

An extensive infectious and rheumatologic evaluation was performed, including testing for tuberculosis, human immunodeficiency virus, Lyme disease, antinuclear antibodies, and rheumatoid factor, all of which were negative (Table 2). A respiratory viral panel from a nasopharyngeal swab returned a positive result for rhinovirus by polymerase chain reaction, identifying it as the most likely etiology.

Based on the clinical presentation, echocardiographic findings, and exclusion of alternative causes, a diagnosis of subacute constrictive pericarditis caused by rhinovirus infection was established. The patient showed progressive symptomatic improvement during hospitalization. A repeat transthoracic echocardiogram obtained two weeks later showed near-complete resolution of the pericardial effusion with normalization of transmitral respiratory variation, disappearance of septal bounce, and restoration of normal lateral mitral annular velocities, indicating resolution of annulus reversus. She was discharged on a tapering course of corticosteroids with continued colchicine and nonsteroidal anti-inflammatory therapy. At the one-month follow-up, she remained free of symptoms with no echocardiographic evidence of recurrent constrictive physiology.

## Discussion

This report highlights a rare but clinically significant complication of rhinovirus infection resulting in subacute constrictive pericarditis. Although rhinovirus is one of the most common viral pathogens worldwide, its cardiac manifestations are infrequently reported and are typically limited to mild myocarditis or acute pericarditis. While multiple cases of rhinovirus-associated pericarditis have been documented, a subacute presentation with constrictive physiology has not previously been described, particularly with detailed assessment using TDI and Doppler flow analysis [10]. This emphasizes the importance for clinicians to consider even common viruses in the differential diagnosis of significant pericardial disease with constrictive features.

The principal diagnostic challenge in subacute constrictive pericarditis lies in its clinical overlap with restrictive cardiomyopathy and other causes of diastolic heart failure. In this setting, echocardiography provides essential hemodynamic insights [11]. Pericardial constraint limits total cardiac volume, leading to exaggerated ventricular interdependence during respiration, which can be detected on Doppler imaging as marked respiratory variation in atrioventricular inflow velocities [11,12]. TDI further improves diagnostic specificity. In constrictive pericarditis, longitudinal myocardial relaxation is preserved, particularly at the septal annulus, due to the tethering effects of the rigid pericardium. This produces annulus reversus, a finding that reliably distinguishes constrictive pericarditis from restrictive cardiomyopathy, in which myocardial relaxation is globally impaired.

The presence of septal bounce reflects abrupt cessation of early diastolic filling due to pericardial constraint and provides additional support for the diagnosis. Importantly, this case represents an inflammatory and potentially reversible phase of constrictive pericarditis rather than chronic fibrocalcific disease. Subacute constrictive pericarditis has been increasingly recognized as a transient condition when identified early and treated appropriately [13]. The favorable outcome in this patient, evidenced by the resolution of pericardial thickening and normalization of Doppler hemodynamics following anti-inflammatory therapy, underscores the critical importance of early diagnosis and intervention. The decision to use corticosteroid therapy was guided by the presence of specific high-risk features: constrictive physiology, large pericardial effusion, and an intense systemic inflammatory response. This approach aligns with management principles for severe inflammatory pericarditis and is supported by literature on transient constrictive pericarditis [11].

The pathophysiology linking an asymptomatic or subclinical rhinovirus infection to severe constrictive pericarditis deserves consideration. Our patient denied preceding respiratory symptoms, suggesting a post-viral immune-mediated process rather than direct viral invasion. Rhinoviruses can elicit potent systemic inflammatory responses, as evidenced by the markedly elevated C-reactive protein level observed in this case [10]. This severe inflammatory state likely triggered pericardial serositis and early fibrin deposition, leading to hemodynamic constraint before mature fibrosis could develop.

While transthoracic echocardiography was sufficient for diagnosis and follow-up in this case, cardiac MRI may provide additional value in selected patients by identifying pericardial edema and late gadolinium enhancement, both indicative of active inflammation and correlating with reversibility [7,3]. Multidisciplinary collaboration among cardiology, infectious disease, and rheumatology teams remains essential to exclude alternative etiologies such as tuberculosis, autoimmune disease, and malignancy.

## Conclusions

Rhinovirus infection should be recognized as a rare but potential cause of subacute constrictive pericarditis. Early recognition is essential, as inflammatory constrictive pericarditis represents a potentially reversible phase of the disease. Comprehensive echocardiographic evaluation, including Doppler assessment of ventricular interdependence, TDI demonstrating annulus reversus, and identification of septal bounce, is central to establishing the diagnosis. Prompt initiation of anti-inflammatory therapy, including corticosteroids in cases with constrictive physiology and significant inflammatory burden, can result in complete clinical and echocardiographic resolution, preventing progression to chronic constrictive disease and the need for surgical intervention.
